# Defunciones por COVID-19: distribución por edad y universalidad de la cobertura médica en 22 países

**DOI:** 10.26633/RPSP.2021.42

**Published:** 2021-04-28

**Authors:** Romain Fantin, Gilbert Brenes-Camacho, Cristina Barboza-Solís

**Affiliations:** 1 Centro Centroamericano de Población, Universidad de Costa Rica San José Costa Rica Centro Centroamericano de Población, Universidad de Costa Rica, San José, Costa Rica.; 2 Facultad de Odontología Universidad de Costa Rica San José Costa Rica Facultad de Odontología, Universidad de Costa Rica, San José, Costa Rica.

**Keywords:** Cobertura de los servicios de salud, infecciones por coronavirus, mortalidad, grupos de edad, Health services coverage, coronavirus infections, mortality, age groups, Cobertura de serviços de saúde, infecções por coronavirus, mortalidade, grupos etários

## Abstract

**Objetivo.:**

Relacionar la distribución etaria estandarizada de las defunciones por COVID-19 en 22 países americanos y europeos, con diferentes indicadores de las características de las poblaciones y de los sistemas de salud.

**Métodos.:**

Las distribuciones de las defunciones por COVID-19 por grupo etario en 22 países americanos y europeos fueron estandarizadas sobre la pirámide de edades de la población mundial. Se calcularon las correlaciones entre la proporción estandarizada de personas de menos de 60 años dentro de las personas fallecidas y cada uno de los seis indicadores.

**Resultados.:**

Se evidenció la existencia de diferencias importantes de distribución por grupo etario entre los países después de haber estandarizado sobre la pirámide de edades a nivel mundial, siendo la proporción de personas de menos de 60 años superior en América Latina y Estado Unidos que en Canadá o Europa occidental. La proporción estandarizada de personas de menos de 60 años dentro de las personas fallecidas por COVID-19 está fuertemente correlacionada con la universalidad de una cobertura médica de calidad (r=-0,92, p<0,01). Esta relación se mantuvo significativa después de haber ajustado sobre los otros indicadores analizados.

**Conclusión.:**

Se propone que las debilidades de la cobertura médica de la población podrían haber creado una mayor letalidad en las poblaciones de menos de 60 años en América Latina y en los Estados Unidos.

## Abstract (en)

**Objective.:**

Relate standardized age distribution of COVID-19 deaths in 22 countries in the Americas and Europe to different indicators of population characteristics and health systems.

**Methods.:**

Distributions of COVID-19 deaths by age group in 22 countries of the Americas and Europe were standardized based on the age pyramid of the world’s population. Correlations were calculated between the standardized proportion of people aged <60 years among the deceased and each of six indicators.

**Results.:**

Standardization based on the world age pyramid revealed considerable differences in age distribution among countries; the proportion of people aged <60 years was higher in Latin America and the United States than in Canada or Western Europe. The standardized proportion of people aged <60 years among persons who died of COVID-19 is strongly correlated to the existence of universal quality medical coverage (r=-0.92, p<0.01). This relationship remained significant after being adjusted for the other indicators.

**Conclusion.:**

We propose that weaknesses in medical coverage of the population may have created higher case-fatality in populations aged <60 years in Latin America and the United States.

## RESUMO

**Objetivo.:**

Correlacionar a distribuição etária padronizada de mortes por COVID-19 em 22 países das Américas e da Europa com diversos indicadores das características das populações e dos sistemas de saúde.

**Métodos.:**

As distribuições das mortes por COVID-19 por faixa etária em 22 países das Américas e da Europa foram padronizadas pela pirâmide etária da população mundial. Foram calculadas correlações entre a proporção padronizada de pessoas com menos de 60 anos entre as pessoas que morreram e cada um dos seis indicadores.

**Resultados.:**

Foram evidenciadas diferenças importantes de distribuição etária entre os países estudados após a padronização pela pirâmide etária da população mundial, sendo maior a proporção de mortes de pessoas com menos de 60 anos na América Latina e nos Estados Unidos que no Canadá ou na Europa ocidental. A proporção padronizada de pessoas com menos de 60 anos entre as pessoas que morreram por COVID-19 está fortemente correlacionada com a universalidade de cobertura médica de qualidade (r=–0,92, p<0,01). Esta correlação se manteve significativa após o ajuste para outros indicadores analisados.

**Conclusão.:**

O nosso estudo sugere que falhas na cobertura médica da população podem ter provocado maior letalidade nas pessoas com menos de 60 anos na América Latina e nos Estados Unidos.

La evidencia acumulada sobre la COVID-19 ha mostrado que uno de los principales factores de riesgo de letalidad de esta patología corresponde a la edad (1, 2). Como resultado, en la mayoría de los países de Europa occidental, únicamente el 5% de las personas fallecidas poseían edades inferiores a los 60 años (3). Sin embargo, en muchos países de América Latina como Brasil, Colombia o Costa Rica, la proporción de personas menores de 60 años que han muerto por causas relacionadas a la COVID-19 supera el 20% del total de las defunciones (4–6) cambiando la imagen de la letalidad del virus en las personas jóvenes y de mediana edad. Intuitivamente, se podría hacer la hipótesis que estas diferencias se deben a la pirámide de edades en cada país. En efecto, en América Latina y el Caribe, el 13% de la población tiene más de 60 años contra el 26% en Europa (7). Sin embargo, es posible que no sea la única explicación. Por ejemplo, en los Estados Unidos, la pirámide de edades es similar a la de Europa, pero el 21% de las personas fallecidas de COVID-19 tenían menos de 65 años, una cifra muy superior a la de Europa (3).

Ahora bien, al contrario de la tasa de mortalidad por COVID-19 cuya relación con las características de los diferentes países ha sido ampliamente estudiada (8), la distribución etaria de las defunciones no depende de manera intrínseca de la magnitud de la epidemia en el país. Por lo tanto, estas diferencias de distribución etaria podrían explicarse bajo dos hipótesis. La primera, es que en los países de Europa occidental, la mortalidad en personas adultas mayores es superior que en países del continente americano. La segunda, es que en los países americanos, la mortalidad asociada a la COVID-19 es mayor en las personas jóvenes y de mediana edad, comparativamente a los países de Europa occidental. Una de las explicaciones posibles de esta segunda hipótesis podría ser la calidad de la atención en salud. En efecto, una atención de baja calidad puede ser particularmente perjudicial para las personas de menos de 60 años, cuya sobrevivencia en los hospitales de Europa occidental es muy alta (9). Si este fuera el caso, una relación entre los indicadores de calidad de los servicios de salud a nivel país y la distribución por grupo etario de las defunciones debería ser observada.

El objetivo de este estudio es el de comparar la distribución de las defunciones por COVID-19 por grupo etario en diferentes países de América y de Europa. Dicha distribución fue comparada con las principales características de salud y del sistema de salud de los diferentes países, lo que permitió emitir hipótesis sobre las causas de dicha distribución.

## MATERIALES Y MÉTODOS

El presente estudio es un análisis ecológico y correlacional, a partir de datos oficiales. Se incluyeron países del continente americano y europeo que contaban con dos tipos de datos. Primero, debían contar con la información sobre la distribución por grupo etario de las defunciones relacionadas a la COVID-19. Segundo, debían poseer las estimaciones de la pirámide de edades en el país. Se excluyeron los países para los cuales la comparación de la mortalidad entre 2020 y los años anteriores demostraron que más de la mitad de las defunciones asociadas a la pandemia de COVID-19 no fueron registradas (México, Perú, Bolivia, Ecuador en particular).

**FIGURA 1.**
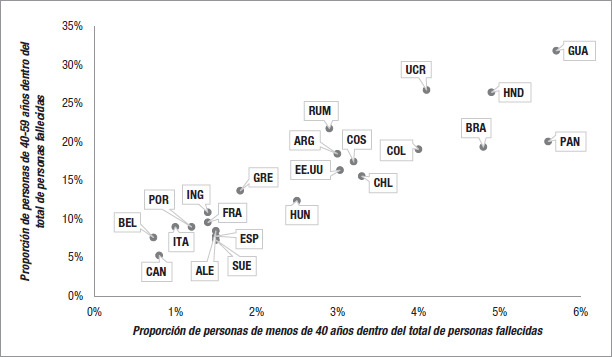
Proporción estandarizada de personas jóvenes (menos de 40 años) y de mediana edad (40-59 años) dentro de las personas fallecidas por COVID-19.

La variable dependiente estudiada es la proporción estandarizada sobre la pirámide de edades de la población mundial de personas jóvenes o de mediana edad, definidas como las personas de menos de 60 años, dentro del total de personas fallecidas por COVID-19. Esta variable se puede dividir entre la proporción de personas de 0 a 39 años (personas jóvenes), y la proporción de personas de 40 a 59 años (personas de mediana edad) (figura 1).

Con respecto a la distribución de las defunciones por COVID-19, los datos corresponden a las defunciones registradas en cada país desde el inicio de la pandemia (marzo 2020) y el periodo comprendido entre el 15 de noviembre y el 5 de diciembre 2020, excepto los datos de España (marzo - 21 de mayo 2020), Brasil (marzo – 24 de octubre 2020), y Portugal (marzo - 15 de agosto 2020).

Los datos que permitieron calcular la distribución de las defunciones por COVID-19 por grupo etario provienen del Ministerio de Salud de Costa Rica (4), de Argentina (5), de Panamá (10), de Chile (11), de Colombia (6), del Ministerio de Salud de Brasil (12), del Ministerio de Sanidad de España (13), de la Universidad Autónoma de Honduras (14), del Ministerio de Salud Pública y Asistencia Social de Guatemala (15), de Estadísticas Canadá (16), de la Organización Pública Nacional (Grecia) (17), del Gobierno de Hungría (18), del Instituto Nacional de Sanidad Pública de Rumania (19), del Instituto Superior de Sanidad (Italia) (20); y de los datos recopilados por el Instituto Nacional de Demográficas (INED por sus siglas en francés) (3) a partir de los datos de *Santé Publique* France (Francia), del Ministerio de Salud (Portugal), del Robert Koch-Institut (Alemania), de la Oficina Nacional de Estadística (Inglaterra y Gales), de los Centros para el Control de Enfermedades (Estados Unidos), de la Agencia de Salud Pública de Suecia (Suecia), del *Sciensano* (Bélgica), del *Ptoukha Institute* (Ucrania) (3).

Según los grupos etarios, en Alemania, Colombia, Costa Rica, España, Francia, Guatemala, Honduras, Hungría, Inglaterra y Gales, Italia, Suecia y Ucrania, se contaba con la distribución de las personas fallecidas por edad decenal, de “0 a 9 años” hasta “90 años y más”. En Brasil y en Canadá, el primer grupo era “0 – 19 años”. En Argentina, Portugal, Canadá, Rumania el último grupo era “80 años y más”. En Chile, el primer grupo era “0 – 39 años”. En Panamá, los datos eran agrupados por edad vicenal.

En Costa Rica y los Estados Unidos, se contaba con la distribución de las personas fallecidas por edad decenal, de “0 a 14 años”, y luego de “15 a 24 años”, hasta “85 años y más”. En Bélgica, se contaba con seis grupos: “0 a 24 años”, “25 a 44 años”, “45 a 64 años, “65 a 74 años”, “75 a 84 años, “85 años y más”. Para estos países, se calcularon la proporción de personas de menos de 65 años (personas de mediana edad), y la proporción de personas de menos de 45 años (personas jóvenes). En Grecia, se contaba con tres grupos: “0 a 39 años”, “40 a 64 años”, “65 años y más”.

En el caso de Francia, para las personas que fallecieron en un hogar de ancianos, solo se contaba con el número de defunciones total (N=9 845). Para estimar la distribución por grupo etario de las defunciones en hogar de ancianos, se utilizaron la distribución por edad de las personas que viven en hogar de ancianos, y la tasa de mortalidad por grupo etario observado en los hospitales.

Los datos que permitieron estimar la pirámide de edades en cada país provienen del Instituto Nacional de Estadísticas y Censo de Costa Rica (21), de Argentina (22), de Panamá (23), del Instituto Nacional de Estadística de España (24), de Chile (25), de Guatemala (26), del Instituto Brasileiro de Geografía y Estadística (27), del Instituto Nacional de Estadística (Rumania) (28), del Instituto Nacional de Honduras (29), de Estadísticas Canadá (30), de los datos recopilados por el INED (Alemania, Bélgica, Francia, Estados Unidos, Italia, Inglaterra y Gales, Portugal, Suecia, Ucrania) (3).

Para evaluar las características de las poblaciones y los sistemas de salud se utilizaron seis indicadores que permitieron aproximar la salud general de las poblaciones y la calidad de la atención en salud en cada país: 1) la esperanza de vida, datos del Banco Mundial para el 2018 (31); 2) diabetes; 3) hipertensión; 4) obesidad (prevalencias estandarizadas por edad de los factores de riesgo de letalidad de la COVID-19 recién mencionados, según datos de la OMS) (32–34); 5) la calidad promedio del sistema de salud fue aproximada gracias al gasto per cápita en dólares (datos del Banco Mundial para el 2017) (35); y 6) la universalidad de la cobertura médica, fue aproximada por el Índice de cobertura efectiva de los servicios de salud calculado por el Grupo de Colaboradores de Cobertura Universal de Salud del Estudio de Carga de la Enfermedad 2019 (GBD)(36). Este índice está construido a partir de 23 indicadores. Cada indicador aproxima el acceso a una atención de calidad a partir de datos de mortalidad o de cobertura de las intervenciones en salud.

Nota: en el caso de los Estados Unidos, de Bélgica y de Grecia, se estimaron las proporciones de menos de 40 y 60 años a partir de las proporciones de menos de 45 y 65 años, y de una regla de tres con los datos de Costa Rica.

ALE:Alemania, ARG:Argentina, BEL:Bélgica, BRA:Brasil, CAN:Canadá, CHL:Chile, COL:Colombia, COS:Costa Rica, ESP:España, EE.UU:Estados Unidos, FRA:Francia, GRE:Grecia, GUA:Guatemala, HND:Honduras, HUN:Hungría, ING:Inglaterra y Gales, ITA:Italia, PAN:Panamá, POR:Portugal, RUM:Rumania, SUE:Suecia, UCR:Ucrania.

***Fuente:*** elaboración propia.

Para el análisis estadístico se estandarizaron las distribuciones sobre la pirámide de edades a nivel mundial (7), utilizando la formula siguiente (37):

$$defunciones_{est,i}=defunciones_{obs,i}*\frac{p_{mundo,i}}{p_{país\;i}}$$

Donde *defunciones_obs,i_* es el número observado de defunciones relacionadas al COVID-19 en el grupo etario *i*, en un país dado; *p_mundo,i_* es la proporción de personas del grupo etario en la población mundial; *p_país,i_* es la proporción de personas del grupo etario i en la población del país analizado.

A partir de las *defunciones_est,i_* estimadas en cada país por cada grupo etario, se calcularon la proporción estandarizada de personas de menos de 40 (o 45) años (personas jóvenes) así como la proporción de personas de menos 60 (o 65 años) en cada país (personas de mediana edad). En el caso Bélgica, Grecia y de los Estados Unidos, se estimaron las proporciones de menos de 40 y 60 años a partir de las proporciones de menos de 45 y 65 años, y de una regla de tres con los datos de Costa Rica, país que contaba con datos por edad quinquenal (cuadro 1).

La correlación entre la distribución por grupo etario y las características de las poblaciones y de los sistemas de salud de los países se hizo utilizando el programa STATA v14, se calculó la correlación de Pearson (r) entre la proporción de personas de menos de 60 años dentro de las personas fallecidas, con respecto a los seis indicadores de las características de las poblaciones y de los sistemas de salud

Este estudio no ha requerido la aprobación de un comité ético científico por tratarse de datos secundarios.

## RESULTADOS

El cuadro 1 presenta las características de las poblaciones y de los sistemas de salud de los países incluidos en el estudio. El cuadro 2, presenta las proporciones observadas de personas jóvenes (menos de 40 o 45 años) o de mediana edad (40-59 años) dentro de las personas fallecidas según los países. También presenta las proporciones de personas jóvenes o de mediana edad (menos de 60 años) estandarizadas sobre la pirámide de edades de la población mundial. La figura 1 evidencia la existencia de tres patrones diferentes de distribución por grupo etario después de haber estandarizado sobre la pirámide de edades a nivel mundial.

Con el supuesto de que los países hubieran tenido la misma pirámide de edades que la población mundial, el primer grupo se refiere a los países en los que menos del 2% de las personas fallecidas por COVID-19 hubieran tenido menos de 40 años, y menos del 13% hubieran tenido menos de 60 años. En este primer grupo, se encuentran Canadá y todos los países de Europa occidental analizados: Alemania, Bélgica, España, Francia, Inglaterra y Gales, Italia, Portugal y Suecia. Nuevamente, con el mismo supuesto, el segundo grupo está compuesto por los países cuyo porcentaje de personas fallecidas por COVID-19 con menos de 40 años está entre 2,9% y 4,0% y cuyo porcentaje de fallecidos con menos de 60 años está entre 18,9% y 24,7%. En este segundo grupo, se encuentran Argentina, Chile, Colombia, Estados Unidos, Costa Rica y Rumania. Dos países se encuentran entre el grupo 1 y el grupo 2: Hungría y Grecia. En un tercer grupo, todavía con el mismo supuesto, entre el 4,1% y el 5,7% de las personas fallecidas por COVID-19 hubieran tenido menos de 40 años, y entre el 24,2 y el 37,6% hubieran tenido menos de 60 años. En este tercer grupo, se encuentran Brasil, Guatemala, Honduras, Panamá y Ucrania.

**CUADRO 1.** Características de los países estudiados de acuerdo a las características de las poblaciones y de los sistemas de salud de los países estudiados.

País	Cobertura médica universal^1^	Esperanza de vida^2^	Gasto en salud per cápita (log)^2^	Prevalencia de diabetes^3^(%)	Prevalencia de obesidad^3^(%)	Prevalencia de hipertensión^3^(%)
Alemania	86	80,9	3,70	5,0	22,3	19,9
Argentina	61	76,5	3,12	9,7	28,3	22,6
Bélgica	87	81,6	3,65	4,6	22,1	17,5
Brasil	65	75,7	2,97	8,3	22,1	23,3
Canadá	90	81,9	3,68	5,5	29,4	13,2
Chile	74	80,0	3,14	10,5	28,0	20,9
Colombia	74	77,1	2,66	8,5	22,3	19,2
Costa Rica	79	80,1	2,94	8,9	25,7	18,7
España	90	83,4	3,40	7,1	23,8	19,2
Estados Unidos	82	78,5	4,01	7,3	36,2	12,9
Francia	91	82,7	3,64	5,9	21,6	22,0
Grecia	80	81,8	3,18	6,6	24,9	19,1
Guatemala	52	74,1	2,41	9,7	21,2	21,2
Honduras	54	75,1	2,29	9,3	21,4	21,4
Hungría	72	76,1	2,99	7,7	26,4	30,0
Inglaterra y Gales	88	81,3	3,59	5,8	27,8	15,2
Italia	89	82,8	3,45	5,8	19,9	21,2
Panamá	71	78,3	3,05	9,3	22,7	19,9
Portugal	84	81,3	3,28	6,8	20,8	24,4
Rumania	70	75,4	2,74	6,8	22,5	30,0
Suecia	90	82,6	3,77	4,9	20,6	19,3
Ucrania	65	71,6	2,25	7,3	24,1	27,1

1 Nota entre 0 y 100, 100 siendo lo mejor. ***Fuente:*** GBD 2019 Grupo de Colaboradores de Cobertura Universal de Salud.

2 En años. ***Fuente:*** Banco Mundial.

3 Prevalencia estandarizada por edad.***Fuente:*** Repositorio de datos del Observatorio Mundial de la Salud

**FIGURA 2.**
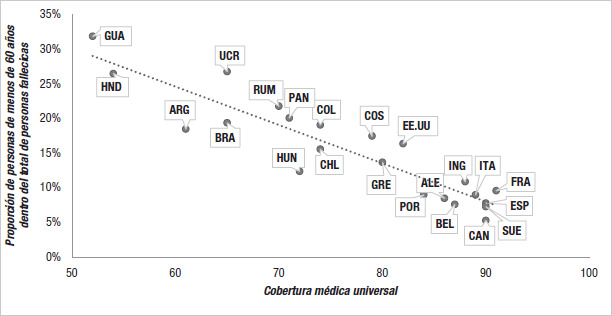
Proporción estandarizada de personas de menos de 60 años dentro de las personas fallecidas por COVID-19 en función de la universalidad de la cobertura médica.

La universalidad de la cobertura médica de calidad está fuertemente correlacionada con la proporción de personas de menos de 60 años dentro de las personas fallecidas (r=-0,92, p<0,01). Los países que tienen una mejor universalidad de la cobertura médica tienen una baja proporción de personas jóvenes o de mediana edad dentro de las personas fallecidas (figura 2). Todos los países de proporción alta o intermediar de personas jóvenes o de mediana edad obtuvieron una calificación inferior al 82/100 (Índice de cobertura efectiva de los servicios de salud), incluyendo los Estados Unidos. Por el otro lado, todos los países con una baja proporción de personas jóvenes o de mediana edad tuvieron una calificación superior al 84 (cuadro 1). Los tres países que tuvieron las calificaciones más bajas fueron Guatemala, Honduras y Ucrania, es decir los tres países con la proporción de personas jóvenes o de mediana edad más alta.

La esperanza de vida (r=-0,88, p<0<01), el gasto en salud per cápita (r=-0,84, p<0,01) y la prevalencia estandarizada por edad de diabetes (r=0,74, p<0,01) se revelaron estar correlacionados con la distribución etaria de las defunciones. Sin embargo, una vez ajustado por la universalidad de la cobertura médica en un modelo de regresión lineal, estas relaciones desaparecieron, lo que no fue el caso de la relación entre universalidad de la cobertura médica que mantuvo significativa (p<0,01). Finalmente, no se encontró una relación estadísticamente significativa entre la distribución etaria de las defunciones y la prevalencia estandarizada por edad de hipertensión (r=0,34, p>0,05) o de obesidad (r=-0,06, p>0,05).

## DISCUSIÓN

La universalidad de una cobertura médica de calidad está fuertemente correlacionada con la distribución etaria de las defunciones. Las características de la población, y en particular la prevalencia de los factores de riesgo, y las otras características de los sistemas de salud de los diferentes países estudiados no permitían explicar dicha correlación. Estos resultados muestran que, a pesar de que la edad es catalogada como uno de los principales factores de riesgo para la mortalidad por la COVID-19, la distribución de las defunciones por grupo etario varía en los diferentes países. Una vez estandarizado sobre una misma pirámide de edades, se mostraron tres grandes grupos de distribución por grupo etario. Un primer grupo con una proporción baja de personas de menos de 60 años en las personas fallecidas (5-13%), que incluía todos los países de Europa occidental y Canadá, pero ninguno de América Latina. Un segundo grupo con una proporción intermedia (18-25%) de personas de menos de 60 años dentro de las fallecidas, que incluía Costa Rica, Argentina, Chile, Colombia, junto con los Estados Unidos y Rumania. Y finalmente, un tercer grupo con una alta proporción de personas de menos de 60 años dentro de las fallecidas (24-38%) que incluía Brasil, Panamá, Honduras, Guatemala, y Ucrania.

De nuestro conocimiento actual, es el primero estudio en el mundo en analizar la relación entre la distribución etaria de las defunciones por COVID-19 y las características de la población y del sistema de salud de los diferentes países. Una explicación de la correlación observada entre la universalidad de una cobertura médica de calidad y la distribución etaria de las defunciones por COVID-19 podría referirse a la calidad de la atención y la existencia de barreras de acceso al servicio de salud para un sector de la población. La calidad de la atención puede resultar en una atención de menor calidad, principalmente en el caso de las condiciones de salud más severas. La existencia de barreras de acceso al servicio de salud puede resultar en una ausencia o un atraso en la atención, y por ende, en una mayor letalidad, principalmente para los sectores menos privilegiados. Esta hipótesis es reforzada por la observación que todos los países del grupo de baja proporción de personas de menos de 60 años poseen una cobertura médica universal del 100% (38). Por el otro lado, dentro de los dos grupos que incluían a todos los países latinoamericanos (grupos 2 y 3), ninguno cuenta con una cobertura médica del 100%. Cabe destacar que, incluso en Costa Rica, que es el país latinoamericano del estudio que tiene el mayor índice de cobertura médica universal, el 15% de la población no está asegurada y esta cifra alcanza el 35% para la población migrante (39). La importancia de una cobertura médica universal de calidad es particularmente relevante en un contexto donde nueve de los diez países americanos estudiados (todos excepto Canadá) presentan un índice de Gini superior a 0,40, lo que refleja importantes desigualdades sociales (40).

**CUADRO 2.** Proporción observada y estandarizada de personas jóvenes (menos de 40 o 45 años) y de mediana edad (40-59 años) dentro de las personas fallecidas por COVID-19.

	N^1^	Menos de 40 años^2^	40-59 años^2^	Menos de 60 años EST^3^
Alemania	14361	0,4%	4,3%	10,0%
Argentina	39156	2,5%	14,4%	21,5%
Brasil	156650	5,3%	21,1%	24,2%
Canadá	12140	0,3%	3,1%	6,1%
Chile	15519	2,3%	13,3%	18,9%
Colombia	37089	4,1%	18,8%	23,1%
Costa Rica	1739	3,6%	17,9%	20,7%
España	20552	0,5%	4,2%	9,3%
Francia	50957	0,4%	4,1%	11,0%
Grecia	2606	0,7%	-	-
Guatemala	4112	9,8%	33,2%	37,6 %
Honduras	2904	7,9%	27,4%	31,4%
Hungría	5142	1,1%	7,3%	14,9%
Inglaterra y Gales	62162	0,6%	5,8%	12,3%
Italia	49930	0,3%	4,3%	10,0%
Panamá	3114	5,6%	20,3%	25,7%
Portugal	1778	0,3%	4,4%	10,2%
Rumania	11331	1,4%	14,8%	24,7%
Suecia	6622	0,5%	3,4%	8,8%
Ucrania	9600	2,5%	20,6%	30,9%

	N^1^	Menos de 45 años^2^	45-64 años^2^	Menos de 65 años EST^3^
Bélgica	16077	0,5%	5,5%	12,4%
Costa Rica	1739	6,4%	26,0%	30,7%
Estados Unidos	240213	2,9%	17,6%	28,8%

			40-64 años^2^	
Grecia	2606	-	13,1%	23,0%

1 Número total de personas fallecidas

2 Distribución observada en el país.

3 Distribución estandarizada sobre la pirámide de edades de la población

Fuente: elaboración propia

La correlación entre una cobertura médica universal de calidad y la proporción de personas de menos de 60 años dentro de las personas fallecidas respalda la hipótesis que indica que la mortalidad fue superior en las personas de menos de 60 años en los Estados Unidos y América Latina, comparativamente a Europa occidental y Canadá. Sin embargo, el subregistro de los casos, en particular asintomáticos o pocos sintomáticos, y las diferentes políticas sobre la realización de pruebas diagnósticas en cada país, no permiten obtener una respuesta clara sobre la distribución de los casos por grupo etario en cada país. Por lo tanto, una de las limitaciones del presente estudio es que no se puede descartar por completo la segunda hipótesis, según la cual la mortalidad fuera superior en las personas adultas mayores en Europa occidental y Canadá comparado a los Estados Unidos, el resto de Europa y América Latina. En efecto, Costa Rica, Argentina, Chile, Europa oriental y algunos estados de los Estados Unidos como Texas o Florida fueron golpeados por la pandemia más tarde que Europa. Esto podría haber permitido a estos países organizar mejor la protección de las poblaciones vulnerables, y en particular las personas adultas mayores. Sin embargo, la robustez de la distribución etaria entre la primera y la segunda ola no respalda esta hipótesis. Por ejemplo, en los Estados Unidos, si bien es cierto que la proporción de personas menores a 64 años fue superior en la segunda ola de la pandemia (16 de mayo – 1ro de octubre, 92 mil defunciones) que en la primera ola (antes del 16 de mayo, 96 mil defunciones) (23% contra 20% respectivamente), la diferencia no es suficiente para explicar la diferencia que observamos con Europa occidental o Canadá (3). Otra explicación podría ser la proporción personas adultas mayores viviendo en hogares de ancianos. En efecto, esta proporción es más importante en Europa que en América Latina y algunos hogares de ancianos se transformaron en foco de contagio. Sin embargo, la proporción de personas adultas mayores en hogares de ancianos varía mucho según los países europeos, siendo por ejemplo mucho más baja en Portugal que en Suecia (41). Si los focos de contagio en hogares de ancianos hubieran permitido explicar la diferencia entre la distribución por grupo etario en Europa y en América Latina, países como Portugal hubieran presentado una distribución intermedia. Finalmente, la última explicación posible se relaciona con la saturación de los servicios de salud, pues en países como Brasil, España, Bélgica, Italia, Inglaterra y Gales, o Francia los adultos mayores posiblemente fueron excluidos del sistema hospitalario en el momento más agudo de la crisis, disminuyendo de facto la calidad de la atención. Sin embargo, en los países europeos menos golpeados por la primera ola como Alemania o Portugal, la proporción de personas de menos de 60 años es similar a la encontrada en los países europeos más golpeados. Finalmente, no existen indicios que permitan respaldar la hipótesis según la cual la diferencia observada de distribución etaria se relaciona con una mayor mortalidad en las personas adultas mayores en los países de Europa occidental y Canadá. Otra limitación del presente estudio es la posibilidad de subregistro de las defunciones asociadas a la COVID-19 en algunos países. Este subregistro podría haber sido superior en las poblaciones de adultos mayores en las cuales la acumulación de comorbilidades puede complicar el diagnóstico de la causa de muerte. Sin embargo, se observaron resultados similares en todos los países de Europa, aunque los estudios de exceso de mortalidad evidenciaron que algunos países (España, Inglaterra y Gales, Italia) habían subestimado la cantidad de defunciones y que otros (Francia, Suecia, Bélgica) no. Además, el análisis del exceso de muertes en Chile no evidenció un subregistro de las defunciones por COVID-19 (42). En España y en los Estados Unidos (43,44), la vigilancia de los excesos de mortalidad evidenció un subregistro de las defunciones por COVID-19, pero sin que la distribución etaria de las defunciones varíe significativamente. Por lo tanto, el subregistro no parece explicar el resultado del estudio.

En el caso de los Estados Unidos, de Bélgica y de Grecia, se estimaron las proporciones de menos de 40 y 60 años a partir de las proporciones de menos de 45 y 65 años, y de una regla de tres con los datos de Costa Rica.

ALE:Alemania, ARG:Argentina, BEL:Bélgica, BRA:Brasil, CAN:Canadá, CHL:Chile, COL:Colombia, COS:Costa Rica, ESP:España, EE.UU:Estados Unidos, FRA:Francia, GRE:Grecia, GUA:Guatemala, HND:Honduras, HUN:Hungría, ING:Inglaterra y Gales, ITA:Italia, PAN:Panamá, POR:Portugal, RUM:Rumania, SUE:Suecia, UCR:Ucrania.

Fuente: elaboración propia.

En conclusión, después de la estandarización sobre la pirámide de edades de la población mundial, se observaron importantes diferencias en la distribución de las personas fallecidas por grupo etario en función de los países. En un grupo intermediario, se encontraron Argentina, Chile, Colombia, Costa Rica, los Estados Unidos y Rumania. La proporción de personas de menos de 60 años era superior a la encontrada en los países de Europa occidental y Canadá, pero inferior a la encontrada en Brasil, Guatemala, Honduras, Panamá, y Ucrania. Se pudo evidenciar una fuerte correlación entre la distribución por grupo etario de las defunciones por COVID-19 y la universalidad de la cobertura médica. El acceso universal a una atención en salud de calidad podría ser un factor clave. Según esta hipótesis, las debilidades de la cobertura médica los países de los grupos 2 y 3 podrían haber creado una mayor letalidad en las poblaciones jóvenes y de mediana edad. Otros estudios a nivel internacional y a nivel local son necesarios para confirmar o infirmar esta hipótesis.

## Declaración.

Las opiniones expresadas en este manuscrito son responsabilidad del autor y no reflejan necesariamente los criterios ni la política de la *RPSP/ PAJPH* y/o de la OPS.
